# Oral Health-Related Quality of Life, Oral Conditions, and Risk of Malnutrition in Older German People in Need of Care—A Cross-Sectional Study

**DOI:** 10.3390/jcm10030426

**Published:** 2021-01-22

**Authors:** Gerhard Schmalz, Clara Rosa Denkler, Tanja Kottmann, Sven Rinke, Dirk Ziebolz

**Affiliations:** 1 Department of Cariology, Endodontology and Periodontology, University of Leipzig, 04103 Leipzig, Germany; Gerhard.schmalz@medizin.uni-leipzig.de; 2 Clinic of General, Special Care and Geriatric Dentistry, Center of Dental Medicine, University of Zurich, 8032 Zurich, Switzerland; clara.denkler@zzm.uzh.ch; 3 Clinical Research Organization, 59063 Hamm, Germany; tk@cro-kottmann.de; 4 Department of Prosthodontics, University Medical Center Goettingen, 37073 Goettingen, Germany; rinke@ihr-laecheln.de; 5 Private Dental Practice, Hanau & Alzenau, 63456 Hanau, Germany

**Keywords:** oral health, nutritional status, nursing home, oral health-related quality of life

## Abstract

Background: The present cross-sectional study assessed oral health, nutritional condition, and oral health-related quality of life (OHRQoL) in older German people in need of care. Methods: The participants were recruited from eight nursing homes (including three nursing homes with assisted living) and one mobile nursing service. Oral health, including dental status (decayed, missing and filled teeth (DMF-T), root caries), periodontal treatment needs, and prosthetic conditions, was recorded. Nutritional status was assessed using the screening of the “Mini Nutritional Assessment” (MNA). The OHRQoL was measured using the German short-form of the Oral Health Impact Profile (OHIP-G14) and summarized as a total sum score as well as the four dimensions “oral function”, “psychosocial impact”, “pain” and “orofacial appearance”. Statistics: Linear logistic regression analyses. Results: A total of 151 participants (age: 84.17 ± 7.8 years) were included. Most participants (60.3%) were nursing home residents. Nearly half of the individuals (47%) were edentulous and 75.4% of the dentate subjects required periodontal treatment. A total of 115 of the subjects had at least one denture. According to the MNA screening, 107 (70.9%) older people were at risk of malnutrition or already suffered from malnutrition. The median OHIP-G14 sum score was 3 (mean 5.7 ± 7.67). Regression analysis revealed MNA to be influenced by DMF-T, D-T, M-T and OHIP G14 sum score and root caries (pi < 0.01). Within the regression model, missing teeth (β: −11.9, CI95: −6.4–−1.9; *p* < 0.01) were the strongest influential factor on MNA, followed by DMF-T (β: 5.1, CI95: 1.7–6.2; *p* < 0.01). Conclusions: Older people in nursing settings show a high prevalence of oral diseases, risk of malnutrition and nearly unimpaired OHRQoL. Dental care should be fostered in these individuals, whereby OHRQoL might be a further hint for increased risk of malnutrition.

## 1. Introduction

Geriatric dentistry is an emerging field. However, a recent systematic map of systematic reviews demonstrated an urgent need for further research in this field [1]. Older people show a variety of oral health concerns, including caries, periodontitis, oral mucosal diseases, edentulism, xerostomia, and temporomandibular joint disorders [1]. The high prevalence of oral diseases and conditions in older people may be due to different reasons. Dynamics in the ecology of the oral cavity and aging-related changes of the tissues and immune response are potentially relevant [2,3]. A low priority of oral health issues in nursing home settings, comorbidities (e.g., diabetes and medication-associated dry mouth) and dementia/cognitive impairment are potential causative factors for oral health deficiencies in older people [4,5]. Therefore, oral conditions in older people, especially if institutionalized, are complex, and strategies to improve their oral care are deemed necessary [5].

Physical oral health, i.e., dental, periodontal and mucosal diseases, affect many aspects of a patient’s life. For example, aspiration pneumonia, which is a frequent cause of death in bed-bound care-dependent older people, can be associated to high oral bacterial load related to dental plaque and oral diseases [6], which may be further complicated due to dysphagia. In addition to the harmful associations between oral and general health in older people, two crucial issues of potential clinical relevance must be mentioned: nutritional status and oral health-related quality of life (OHRQoL). Poor oral health might be related to a risk of malnutrition or malnourishment in older people, especially if they are institutionalized [7,8,9,10]. This might be explained by oral complaints such as pain, tooth loosening or extended tooth loss leading to the impairment of chewing and swallowing food. However, a recent meta-analysis concluded that more research is necessary to clarify the influence of oral conditions on the nutritional situation [9].

Beside physical oral health, the OHRQoL provides insight into the patients’ perception of their oral conditions and constitutes a patient-reported outcome of clinical importance. OHRQoL in older people is associated with poor oral conditions and disease-specific parameters, such as dementia, depression and dry mouth [11,12,13,14,15], as well as the nutritional status [16]. Other studies demonstrated that low nutritional status was associated with worse OHRQoL [17,18,19]. Thereby, the literature is quite heterogeneous, e.g., regarding associations between dementia and OHRQoL [11]. The relationship between oral health, OHRQoL, and the risk for malnutrition is of high practical relevance, and should be considered to improve the dental care of patients.

To understand the value of the OHRQoL’s outcomes, the results may be interpreted in four dimensions, including oral function, psychosocial impact, orofacial pain, and orofacial appearance [20,21]. However, these dimensions were not considered in previous studies. The purpose of the current cross-sectional study was to assess the relationship between oral health, nutritional condition, and OHRQoL in older German people in need of care. The hypothesis set for the current study was that the OHRQoL of elders would be associated with poor oral health and a risk for malnutrition.

## 2. Methods

The current study was a multicenter cross-sectional study. The study received ethics approval from the ethics committee of the medical faculty of the University of Leipzig (No. 079-16-14032016). A single dentist (CRD) previously informed eight nursing homes as well as one mobile nursing service about the study. All of them agreed to participate and signed their formal written informed consent. The participants and/or their legal guardians were also informed (see below) and gave their written informed consent, too. The study was conducted according to the guidelines formulated in the Declaration of Helsinki.

### 2.1. Patients

Participants were recruited from eight nursing homes (including three nursing homes with assisted living) and one mobile nursing service that visited participants at home. All of the institutions were localized in the county of Schwäbisch Hall, Baden-Wuerttemberg, Germany. It was estimated, that about 500 older people would be in care in the respective institutions or mobile services. With regard to the in- and exclusion criteria and an expected participation rate of 30%, a case number of 150 older people was estimated and aspired towards. To inform the participants or legal guardians previously and ask for their consent for participation, numerous individual methods were used—e.g., personal information from the study dentist or written information about the study. Only subjects who voluntarily agreed to participate (himself or herself or informed consent by the legal guardian) were included. The following inclusion criteria were used for participation in the study: resident in a nursing home or assisted living facility, or using a mobile nursing service, being female or male, and aged at least 60 years. Exclusion criteria were lack of cooperation or the inability to undergo oral examinations.

### 2.2. Recording of Subject Data

The participants’ health records from the nursing home/assisted living or mobile nursing facility were screened for general and medical data whenever possible, otherwise participants (in some cases also accompanying and supporting relatives) were asked for this information in personal interviews carried out by the study dentist. The following data were recorded: age, gender, smoking status, dementia or general diseases such as diabetes mellitus or rheumatic diseases, living conditions (nursing home, assisted living or at home using mobile nursing), and resilience as described by Nitschke et al. [22]. No additional tests, e.g., grade of dementia severity, were performed.

### 2.3. Assessment of Nutritional Condition

For the assessment of nutritional status, only the screening of the “Mini Nutritional Assessment” (MNA^®^) was used [23,24]. The full assessment consists of different measurements (weight, height, and weight loss in previous three months) combined with brief questions related to lifestyle, medication, mobility, diet and subjective assessment. Based on weight and height, the Body Mass Index (BMI) was calculated. The results were documented in a point score. The maximum for the applied screening is 14 points. A score ≥ 12 indicates an adequate nutritional status, a score between 8 and 11 shows a risk of malnutrition, and a score ≤ 7 denotes malnutrition [23].

### 2.4. Assessment of Oral Health-Related Quality of Life

OHRQoL was assessed using the German short version of the Oral Health Impact Profile (OHIP-G14), which is a valid instrument for OHRQoL assessment [25,26,27]. This questionnaire collected 14 functional and psychosocial impacts that the participants experienced in the previous month as a result of their dental problems (teeth, mouth, and dentures). The optional answers for each question are given on a five-point scale between 0–4 as follows: very often = “4”, fairly often = “3”, occasionally = “2”, hardly ever = “1”, and never = “0”. Therefore, a higher score reflects worse OHRQoL. For interpretation and further analysis, the sum score of the OHIP-G14 and the four different dimensions, “oral function”, “psychosocial impact”, “oral pain”, and “orofacial appearance”, were considered [20].

### 2.5. Oral Examination

A single dentist (CRD) performed the dental examinations of all included participants under standardized conditions and collected the data. The investigation included dental findings (decayed, missing and filled teeth, as well as root caries), periodontal examination, prosthodontic situation (form and sufficiency of dentures), and inspection of the oral mucosa.

Dental findings: To assess the dental status, the decayed, missing and filled teeth index (DMF-T) was assessed visually using a mirror and probe. All teeth with reasonable suspicion of/definitely showing a cavitation in the dentine layer, as well as filled teeth being decayed, were assigned to the D (= decayed) component. Filled and crowned teeth were assigned to F (= filled), and missing teeth were assigned to the M component (= missing). Wisdom teeth were not examined, and a maximum score of 28 could be reached [28]. The oral cavity was scanned for residual roots.

Periodontal screening: The periodontal situation was evaluated using the periodontal screening index (PSR^®^/PSI), which reflects the need for periodontal treatment. For this index, a WHO probe (Henry Schein dental GmbH, Langen, Germany) was used for periodontal probing at 6 points per tooth. The following interpretation criteria were used:

- Code 0—pocket depth < 3.5 mm, no bleeding, and no calculus;
- Code 1—pocket depth < 3.5 mm, bleeding on probing, and no calculus;
- Code 2—pocket depth < 3.5 mm, bleeding on probing, and calculus is present;
- Code 3—pocket depth is 3.5–5.5 mm;
- Code 4—pocket depth is >5.5 mm.

The jaw was divided into sextants, each with three sections (one anterior and two posterior tooth segments) in the upper and lower jaw. The highest score was determined for each sextant. A PSR^®^/PSI score of 3 or 4 was interpreted as requiring periodontal treatment [29,30].

Prosthodontic situation: Fixed dental prostheses (e.g., crowns) were recorded for sufficiency within the DMF-T index (F-T component or D-T component if decayed). For removable dental prostheses, whether it was a partial or total prosthesis was recorded for each jaw separately. The dentures were classified into sufficient or insufficient following clinical criteria (e.g., precise fit of the denture, condition of prosthetic teeth, and functional parameters).

### 2.6. Statistical Analysis

Statistical analyses were performed using SPSS for Windows (SPSS Inc., Stanford, CA, US). All metric variables were tested using the Kolmogorov–Smirnov test for normal distribution, and no parameters were normally distributed. Therefore, nonparametric tests for non-normally distributed samples were used. Descriptive data are presented as the means ± standard deviation or *n* (percentage). To assess the influence of different, independent variables over MNA and OHIP-G14 results, a linear regression analysis was used, whereby the groups were determined by median values. Prior to regression analysis, variables were tested by ANOVA, whereby regression analysis was only executed in the case of statistical significance. Within regression analysis, the influence of different independent variables on the dependent variable was analyzed. In the applied analysis the MNA total sum score was the dependent variable, which was analyzed with regard to the independent variables dementia, DMF-T, D-T, M-T, root caries, residual roots, denture wearing and OHIP-G14 sum score. The significance level was set at *p* < 0.05.

## 3. Results

### 3.1. Patients

In total, 462 older people were asked for their voluntary participation. Thereby, 170 gave their informed consent. Out of these patients, 19 were excluded from analysis because of not meeting the in- and exclusion criteria (*n* = 7), withdrawal of consent (*n* = 4), severe general disease (*n* = 7) or death (*n* = 1), respectively. Accordingly, 151 participants (participation rate of 32.7%) with a mean age of 84.17 ± 7.8 years were included in this study. Most participants (60.3%) were institutionalized (nursing home residents). Dementia was prevalent in more than one-third of the participants (37.1%). Only 10 (6.6%) participants had a normal resilience. Further participants’ characteristics are presented in Table 1.

### 3.2. Nutritional Condition (MNA)

The overall mean screening MNA score was 10.1 ± 2.4 for the total cohort. Most participants, i.e., 76.8% had a BMI of 23 or higher. A total of 107 (70.9%) of the included individuals were at risk for malnutrition or already suffered from malnutrition (Table 2).

### 3.3. Oral Health Findings

The average DMF-T of the total cohort was 26.41 ± 3.04. Edentulism was present in about 47% of the participants. Periodontal treatment need of 75.4% was obvious in the periodontally examined individuals (*n* = 69). A total of 115 of the subjects had at least one denture, and 52.7 % of the upper jaw prostheses and 73.7% of the lower jaw prostheses were insufficient (Table 3).

### 3.4. Oral Health-Related Quality of Life

The median of the OHIP-G14 sum score was 3 (mean 5.7 ± 7.67). For the dimension oral function, a median of 2 (mean 3.01 ± 3.47) was found. The other three dimensions, psychosocial impact (mean 1.52 ± 3.45), oral pain (mean 0.8 ± 1.27), and orofacial appearance (mean 0.38 ± 0.99), had a median of 0 (Table 4).

### 3.5. Logistic Regression Analysis

For MNA as the dependent variable, ANOVA revealed statistical significance (R^2^ = 0.23, *p* < 0.01). Within the subsequent regression model, the MNA score was most strongly influenced by M-T (β: −11.9, CI95: −6.4–−1.9; *p* < 0.01), followed by DMF-T (β: 5.1, CI95: 1.7–6.2; *p* < 0.01), D-T (β: −3.1, CI95: −6.2–−1.6; *p* < 0.01), the presence of root caries (β: −0.3, CI95: −1.3–−0.2; *p* < 0.01) and the OHIP-G14 sum score (β: −0.2, CI95: −0.1–0.0; *p* = 0.05; Table 5). For the different dependent variables OHIP G14 sum score (R^2^ = 0.10, *p* = 0.16), oral function (R^2^ = 0.11, *p* = 0.08) and psychosocial impact (R^2^ = 0.07, *p* = 0.40), ANOVA did not find significance. Therefore, no further regression analysis was executed for these variables.

## 4. Discussion

To interpret the oral health findings of the current study, the Fifth German Oral Health Study (DMS V) was used as a population-representative cohort for comparison in the absence of a healthy control group [31]. A previous study by some authors of this working group also found increased caries prevalence, periodontal treatment need, and a fairly similar number of edentulous individuals (48% vs. 47%) compared to the current study [7]. As presented in a recent systematic review, the international literature showed the number of decayed teeth between 1.2 and 3.5, which is slightly higher than that in the current study, of 1.06 [4]. The need for periodontal treatment is high in dentate older people (DMS V). This result is consistent with the findings of the current study [4]. However, it needs to be mentioned that periodontal examination was only possible in 69 older people (45.7%), which is a potential bias in favor of compliant, healthy and non-demented participants. A Belgian study that also used the PSI score found a need for periodontal treatment of 73%, which was comparable with the current study [32]. In the institutionalized older people, a range of 20.4% to 62% for edentulism was reported [4]. A total of 47% of edentulous individuals are within this range, and this seems consistent with the international literature. Overall, the oral health findings of the cohort in the current study were similar to the available German and international findings.

Older people have an increased risk of malnutrition, and screening for this issue is reasonable in these individuals [33]. Age and level of care are important factors that heighten the risk, especially for protein-energy malnutrition [34]. Therefore, the high risk in the current study is consistent with the literature—more than 70% of the individuals in the current study were at risk for malnutrition or already malnourished according to the MNA. The previous study found a lower prevalence for the risk of malnutrition in nursing home residents (52%), but a comparable mean value for the MNA screening points [7]. As influential factors on MNA, being edentulous and dementia were the risk indicators in the previous study [7]. Missing teeth were the strongest influential factor for malnutrition in the regression analysis within the current study, and dementia only tended to influence MNA. The results of a systematic review with meta-analysis confirmed that the number of teeth was associated with nutritional status in older people [9]. Recent studies also reported that caries and/or toothache while chewing increased the risk of malnutrition in this patient group [35,36,37]. This result appears consistent with the findings of the current study, which found that decayed teeth and root caries were influential factors for MNA. Therefore, it must be recognized that the influence of root caries on MNA was just small. Dementia increases the risk for malnutrition in older people [38,39], and it is somewhat surprising that dementia only tended to be a risk indicator for malnutrition in the current study.

The current study used OHIP-G14, which is a validated instrument for the assessment of OHRQoL [26]. Whether the Geriatric/General Oral Health Assessment Index (GOHAI) would have been more appropriate for the cohort of the current study is debatable because it is necessary to choose the appropriate instrument for older people [40]. However, OHIP-G14 was chosen in the present study because the questions are easily assigned to the four dimensions, according to John et al. [20]. OHIP-14 and GOHAI are strongly correlated with each other [40,41]. Reference values are available for OHIP-G14, which may be used in the absence of a healthy control group [42]. The scale for the reference depends on the dentition range between 0 (fully dentate) and 6 points (edentulous, wearing total prostheses) [42]. Considering that nearly half of the participants in the current study were edentulous, the study population had a sum score within this range (median 3, mean 5.7). Therefore, the OHRQoL as assessed with OHIP-G14 in the current study appeared comparable to the German general population. Regression analysis was omitted from the OHIP G14 findings in the absence of significance in ANOVA for the chosen parameters. These findings are not consistent with recent studies that found a compromised OHRQoL in the older people, which was predicted by dental problems and/or missing teeth [12,13,14]. However, these studies regularly used GOHAI. This result suggests a more appropriate correlation of this measurement in the older people cohort compared to the OHIP-G14 that was used in the present study. The hypothesis set for the current study was that the OHRQoL of older people would be associated with poor oral health and a risk for malnutrition. It has been repeatedly reported that poor oral conditions, especially tooth complaints, tooth wear, missing teeth and denture wearing, as well as sufficiency of dentures, are important influential factors for OHRQoL [43,44,45,46]. Therefore, the first part of the hypothesis appears reasonable. The findings of the current study primarily found an effect in the dimension of oral function of OHRQoL in older people. This finding suggests an influence of physical oral conditions on the OHRQoL of the individuals in the present study. However, multivariate analysis was not able to confirm this in the current study. A missing influence of the physical oral parameters on OHRQoL might be caused by the choice of inappropriate oral complaints for analysis, e.g., xerostomia or burning mouth syndrome, which are influential factors on the OHRQoL in older people [40]. The influence of OHRQoL on MNA may be clinically relevant and confirms the study hypothesis. However, the magnitude of the influence of OHIP-G14’s findings on MNA was small within the model, which needs to be considered in the interpretation of the findings and makes the practical importance a little questionable. Appropriate nutrition is positively associated with OHRQoL, and a low nutritional status would be related to worse OHRQoL [16,17,18]. A randomized controlled trial showed that dietary advice and prosthetic treatment improved nutrition and OHRQoL in older people [19]. Therefore, a strong relationship between nutrition and OHRQoL seems conceivable for these individuals, which was confirmed in the present study, although the effect size in the present study was relatively small (β −0.2). Overall, these findings indicate that older people in a nursing setting need improved oral care, and patients with chewing problems and/or discomfort while eating should receive increased attention to decrease their risk for malnutrition.

### Strengths and Limitations

This is the first study to investigate the relationship between OHRQoL and its dimensions with physical oral health and nutritional status in older Germans who require nursing assistance (nursing home, assisted living or use of a mobile nursing service). The sample size seemed appropriate. However, the group was quite heterogeneous (different nursing settings, mental status, oral conditions). Several methodological issues must be addressed. Overall, the GOHAI could have been performed additionally to ensure that the OHIP-G14 provided appropriate information on the OHRQoL of the participants. The study design was very extensive, because of the examination and the surveys. Thus, a further questionnaire such as GOHAI would have placed too much strain or burden on the participants and possibly led to confusion. The OHIP G14 was chosen for several reasons: first, the four dimensions as presented by John et al. [20] can be analyzed separately, which helps us to interpret the OHRQoL of the participants. Second, reference values for the general population are available, making a comparison to these values with general population possible. Third, the OHIP G14 has been shown to be an appropriate tool for research questions of many different populations [21]. The absence of a comparison group may be considered as a limitation. However, reference values for oral health (DMS V, [21]) and OHIP-G14 [42] are available and were discussed accordingly. Information with a high relevance for OHRQoL and nutritional condition, e.g., xerostomia, dysphagia, burning mouth, etc., were not assessed and should be considered in future studies. Generally, the consideration of oral health parameters in the current study is limited, because, especially in older people, a variety of intra- and extra-oral symptoms and complaints, including tooth wear, temporomandibular diseases, mucosal diseases or functional problems might be of relevance. Furthermore, several parameters, including age, level of care and denture status (fixed dental prosthesis, removable denture, total denture) might be further influential parameters on the study’s results. To strengthen the power of the analysis in this current study, these parameters were not included in the regression model. This must be recognized as a limitation of the study. The cross-sectional design did not allow an interpretation of risk predictors. A causative relationship could only be assessed in a prospective setting. Although the MNA is a valid questionnaire to assess risk of malnutrition in older people, its significance is limited in patients with cognitive impairment [43]. Therefore, the results for the demented study participants are limited by this fact. Altogether, the current study provides some findings of clinical relevance. However, the value of these results for the improvement of dental care in the older people must be confirmed in further studies.

## 5. Conclusions

Older people in nursing settings show a high prevalence of oral diseases, need for periodontal treatment, and risk of malnutrition. The OHRQoL was nearly unimpaired, but influenced the risk of malnutrition, alongside physical oral health, whereby missing teeth were the strongest influential factor. Therefore, oral care should be fostered in these individuals to reduce the risk for malnutrition. Thereby, a perceived impairment in OHRQoL could be a further hint for an increased risk of malnutrition. Particular attention should be paid to patients with complaints while eating to ensure their nutritional condition and OHRQoL.

## Figures and Tables

**Table 1:** Participants’ characteristics (*n* = 151).

**Age in Years (mean ± SD)** **(median; range)**		84.17 ± 7.8 (85.0; 62–99)
**Gender**	female	104 (68.9%)
	male	47 (31.1%)
**Current smoker**	nonsmoker (includes nonsmokers and former smokers)	140 (95.9%)
	smoker	6 (4%)
**Dementia**	no	95 (62.9%)
	yes	56 (37.1%)
**Diabetes mellitus**	no	92 (60.9%)
	yes	59 (39.1%)
**Rheumatic diseases**	no	79 (52.3%)
	yes	72 (47.7%)
**Living conditions**	nursing home	91 (60.3%)
	assisted living	20 (13.2%)
	using mobile nursing service at home	40 (26.5%)
**Resilience**	normal	10 (6.6%)
	slight reduction	30 (19.9%)
	strong reduction	70 (46.4%)
	no	41 (27.2%)

SD: standard deviation.

**Table 2:** Results of the MNA (Mini Nutritional Assessment) screening of the participants (*n* = 151). Only the first part (=screening) of the MNA was performed for the participants.

**Declined Food Intake Over the Past 3 Months**	severe decrease	14 (9.3%)
	moderate decrease	27 (17.9%)
	no decrease	110 (72.8%)
**Weight Loss During the Past 3 Months**	>3 kg	15 (9.9%)
	1–3 kg	38 (25.2%)
	no	91 (60.3%)
	unknown	7 (4.7%)
**Mobility**	bed- or chair-bound	48 (31.8%)
	able to get out of bed/chair but does not go out	34 (22.5%)
	goes out	69 (45.7%)
**Psychological Stress or Acute Disease During the Past 3 Months**	no	100 (66.2%)
	yes	51 (33.8%)
**Neuropsychological Problems**	no psychological problems	62 (41.1%)
	mild dementia	17 (11.3%)
	severe dementia or depression	72 (47.7%)
**Body Mass Index** **(kg/m^2^)**	BMI < 19	2 (1.3%)
	19 ≤ BMI < 21	8 (5.3%)
	21 ≤ BMI < 23	25 (16.6%)
	>23 BMI ≥ 23	116 (76.8%)
**MNA Screening Points (Mean ± SD)**		10.1 ± 2.4
**Nutritional Status**	normal nutritional status	44 (29.1%)
	at risk of malnutrition or already suffers from malnutrition	107 (70.9%)

SD: standard deviation.

**Table 3:** Oral health of the participants and comparable data from the representative population in the fifth German Oral Health Study (DMS V) [31].

Parameter			Current Study (Mean ± SD) (Median)	German General Population (DMS V) Age 65–74 Years	German General Population (DMS V) Age 75–100 Years	German General Population (DMS V) Age 75–100 Years in Need of Care
All participants (*n* = 151)	DMF-T index		26.41 ± 3.04 (28)	17.7	21.6	24.5
	decayed teeth		1.06 ± 2.30 (0)	0.5	0.6	0.7
	missing teeth		22.52 ± 7.20 (27)	11.1	17.8	22.4
	filled teeth		2.84 ± 4.39 (0)	6.1	3.2	1.4
	root caries		0.41 ± 1.07 (0); 19.2%	28%	26%	18%
	residual roots		0.45 ± 1.70 (0)	-	-	-
	edentulous		71 (47.0%)	12.4%	32.8%	62.1%
Dentate participants who underwent periodontal probing (*n* = 69)	periodontal treatment need		52 (75.4%)	75.4%	80.6%	70%
	PSI/PSR max	Score 1	1 (1.4%)	24.6% *	19.4% *	30% *
		Score 2	16 (23.2%)			
		Score 3	34 (49.3%)	50.8% *	50.5% *	35.1% *
		Score 4	18 (26.1%)	24.6% *	30.1% *	34.9% *
Prosthodontic care situation	edentulous without dentures		2 (1.3%)	Prosthesis upper and lower jaw: 28.7% Prosthesis upper jaw: 10.9% Prosthesis lower jaw: 6.2% Thereof total prosthesis: 43.1%	Prosthesis upper and lower jaw: 55.5% Prosthesis upper jaw: 12.4% Prosthesis lower jaw: 3.9% Thereof total prosthesis: 58.8%	Prosthesis upper and lower jaw: 73% Prosthesis upper jaw: 9.7% Prosthesis lower jaw: 3.0% Thereof total prosthesis: 67.6%
	total prosthesis upper jaw		88 (58.3%)			
	partial prosthesis upper jaw		24 (15.9%)			
	prosthesis upper jaw insufficient		59 (52.7%)			
	total prosthesis lower jaw		51 (33.8%)			
	partial prosthesis lower jaw		44 (29.1%)			
	prosthesis lower jaw insufficient		70 (73.7%)			

SD: standard deviation, PSI: periodontal screening index, PSR: periodontal screening record. * instead of PSI/PSR, the community periodontal index (CPI) was used in the DMS V.

**Table 4:** Oral health-related quality of life of the participants as assessed using the OHIP-G14. Some of the participants (especially with severe dementia) were not able to complete the questionnaire and were handled as missing values (see *n*).

OHIP-G14 Dimensions/Questions	OHIP-G14 Scores					
	0	1	2	3	4	Total Mean ± SD (Median)
**Oral Function**						
Total oral function	-					3.01 ± 3.47 (2.0)
Trouble pronouncing words (*n* = 133)	103	11	11	5	3	0.45 ± 0.95 (0)
Sense of taste worsened (*n* = 127)	116	0	7	1	3	0.23 ± 0.79 (0)
Interrupt meals (*n* = 132)	102	11	12	5	2	0.44 ± 0.92 (0)
Uncomfortable eating (*n* = 134)	49	25	34	12	14	1.38 ± 1.33 (1)
Diet unsatisfactory (*n* = 128)	95	12	6	10	5	0.58 ± 1.13 (0)
**Psychosocial Impact**						
Total psychosocial impact	-					1.52 ± 3.45 (0)
Life less satisfying (*n* = 127)	96	9	16	2	4	0.50 ± 0.99 (0)
Difficulty relaxing (*n* = 127)	110	4	8	2	3	0.30 ± 0.85 (0)
Felt tense (*n* = 127)	111	7	5	1	3	0.25 ± 0.77 (0]
Irritable with other people (*n* = 127)	121	3	2	1	0	0.08 ± 0.39 (0)
Difficulty doing usual jobs (*n* = 126)	118	2	4	2	0	0.13 ± 0.52 (0)
Unable to function (*n* = 126)	123	1	1	1	0	0.05 ± 0.33 (0)
Been embarrassed (*n* = 129)	114	3	4	3	5	0.31 ± 0.94 (0)
**Oral Pain**						
Painful aching in mouth (*n* = 136)	90	10	20	6	10	0.8 ± 1.27 (0)
**Orofacial Appearance**						
Felt self-conscious (*n* = 130)	109	6	7	2	6	0.38 ± 0.99 (0)
**OHIP-G14 Sum Score**						
Sum score	-					5.7 ± 7.67 (3.0)

SD: standard deviation; some participants did not answer all the questions; for each question, the number of patients who answered this question is provided.

**Table 5:** Regression analysis of MNA as dependent variable for different independent parameters.

Parameters	β	CI_95_ Lower	CI_95_ Upper	*p*-Value
Dementia	−0.1	−1.6	0.1	0.06
DMF-T	5.1	1.7	6.2	<0.01
D-T	−3.1	−6.2	−1.6	<0.01
M-T	−11.9	−6.4	−1.9	<0.01
Root caries	−0.3	−1.3	−0.2	<0.01
Residual roots	−0.2	−1.0	0.2	0.16
Denture wearing	0.1	−0.8	1.6	0.48
OHIP-G14 sum score	−0.2	−0.1	0.0	0.05

## Data Availability

The data presented in this study are available on request from the corresponding author.
